# Assessing changes in aortic motion and hemodynamics after valve-sparing aortic root surgery in Marfan syndrome using four-dimensional balanced steady-state free precession and four-dimensional flow cardiovascular magnetic resonance

**DOI:** 10.1016/j.jocmr.2026.102728

**Published:** 2026-04-17

**Authors:** Daan Bosshardt, Renske Merton, Eric M. Schrauben, Aart J. Nederveen, Moniek G.P.J. Cox, Daniëlle Robbers-Visser, Dave R. Koolbergen, Maarten Groenink, Pim van Ooij

**Affiliations:** a Radiology and Nuclear Medicine, Amsterdam University Medical Center, Amsterdam, the Netherlands; b Amsterdam Cardiovascular Sciences, Amsterdam, the Netherlands; c Cardiology, Amsterdam University Medical Center, Amsterdam, the Netherlands; d Cardiology, University Medical Center Groningen, Groningen, the Netherlands; e Cardiothoracic Surgery, Amsterdam University Medical Center, Amsterdam, the Netherlands

**Keywords:** Marfan syndrome, Aortic surgery, 4D CMR, Hemodynamics, Biomechanics

## Abstract

**Background:**

Aortic root surgery in patients with Marfan syndrome (MFS) has significantly improved survival, allowing time for distal aortic complications such as type B aortic dissection (TBAD) to emerge. The implantation of a non-compliant synthetic graft may alter aortic biomechanics and hemodynamics, potentially contributing to these late complications. This proof-of-concept study aimed to assess changes in aortic motion and flow characteristics following aortic root surgery using advanced cardiovascular magnetic resonance (CMR) techniques.

**Methods:**

Three MFS patients (aged 26–37 years), two males, one female, undergoing valve-sparing aortic root surgery, were prospectively studied before and 6 months after surgery. All participants underwent non-contrast-enhanced four-dimensional (4D) balanced steady-state free precession and 4D flow CMR at 3T. A deep learning–based segmentation pipeline (nnU-Net) provided segmentations utilized for calculation of aortic displacement, distensibility, regional wall shear stress (WSS) and velocity, and global pulse wave velocity (PWV).

**Results:**

Postoperatively, all patients exhibited markedly decreased ascending aorta (AAo) volume. Three-dimensional distensibility (10⁻³ mmHg⁻¹) also decreased: patient 1, from 4.1 to 1.7; patient 2, from 3.4 to 1.2; and patient 3, from 2.9 to 1.5. Displacement in the AAo, especially at the sinotubular junction, was substantially reduced, consistent with the rigidity of the implanted graft. In the descending aorta (DAo), distensibility and displacement showed increases in two patients and a decrease in one. Peak systolic velocity and WSS increased in the AAo for all patients, whereas DAo values remained largely unchanged. PWV change varied between patients: decreasing slightly in patient 1, from 8.4 to 7.9 m/s, but increased in patients 2 and 3 (8.0 to 8.9 m/s and 6.2 to 9.8 m/s, respectively.

**Conclusion:**

This study demonstrated that AAo biomechanics and hemodynamics change predictably following valve-sparing aortic root surgery in MFS, while changes in the DAo were not consistent between patients. Further research with larger sample sizes is required to identify which of these changes are linked to specific disease profiles and whether parameter combinations can indicate a predisposition to TBAD.

## Graphical abstract

**Figure:**
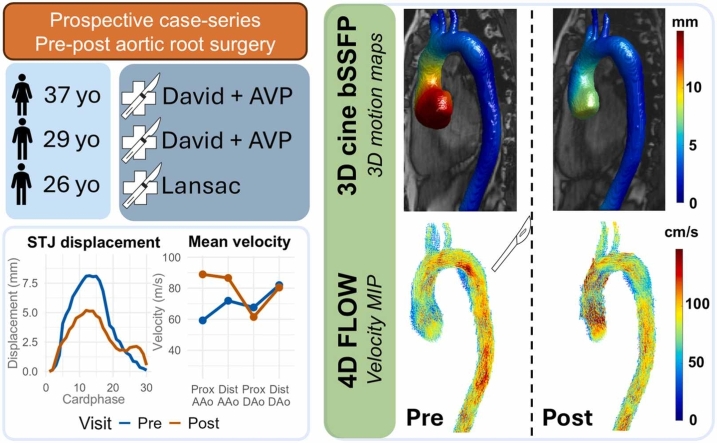

## 1 Introduction

Aortic root surgery to treat thoracic aortic aneurysms in patients with Marfan syndrome (MFS) has led to increased life expectancy [1]. This longer survival allows more time for other aortic complications to develop, with type B aortic dissection (TBAD) emerging as a growing concern in MFS [2]. The implantation of a non-compliant aortic graft may induce changes in aortic biomechanics and intra-aortic hemodynamics, potentially contributing to the development of TBAD. Therefore, it is important to investigate whether, and to what extent, these parameters change after surgery.

Four-dimensional (4D) flow cardiovascular magnetic resonance (CMR) imaging has been extensively used to evaluate aortic hemodynamics and pulse wave velocity (PWV), a proxy for aortic stiffness, in MFS. We have recently developed a non-contrast-enhanced three-dimensional (3D) cine balanced steady-state free precession (4D bSSFP) CMR sequence for 3T, along with a deep learning–based automatic segmentation workflow [3]. This approach enables visualization and quantification of 3D motion in the thoracic aorta with good repeatability, and we have previously demonstrated that 3D distensibility and displacement are abnormal in patients with MFS [3], [4]. The aim of this proof-of-concept longitudinal study was to assess patient-specific pre-to-post surgical changes in aortic motion and hemodynamics.

## 2 Methods

For this prospective pre- and post-surgery case series, patients were identified in two Dutch hospitals with a specialized multidisciplinary Marfan screening clinic. All patients underwent CMR before and 6 months after valve-sparing aortic root surgery, using either the David (replacement) or Lansac (remodeling) technique. The local ethics boards approved the study, and written informed consent was obtained from all participants. All participants underwent imaging of the thoracic aorta on a 3T scanner (Ingenia, Philips Healthcare, Best, the Netherlands) equipped with a 16-channel anterior coil and 12-channel posterior coil. Non-contrast enhanced, free-breathing 4D bSSFP and 4D flow MRI, the same field of view (FOV) was used with an in-house developed pseudo-spiral Cartesian acquisition scheme and reconstructed using a compressed sensing pipeline [3], [4]. Scan parameters are presented in Table 1. For both scans, the cardiac signal was extracted using either electrocardiography or photoplethysmography. Velocity encoding for 4D flow CMR was 150–175 cm/s. All velocity data were automatically corrected for background phase offsets and velocity wrapping (reference Loecher et al.) during reconstruction [5], [6]. Time-averaged phase-contrast magnetic resonance angiogram images (phase-contrast magnitude images multiplied by absolute velocity) were created [5]. A clinical mDixon scan was used to determine presurgical maximal cusp-to-cusp root diameters.

**Table 1:** Scan settings for 4D bSSFP and 4D flow CMR scan.

Scan parameter	Scan type	
	4D bSSFP	4D flow CMR
TE/TR/FA (ms/ms/°)	2.9/1.4/40°	4.7/2.5/4°
FOV (mm³)	256 × 256 × 70-88	256 × 256 × 70-88
Slice oversampling	1.7–2.1	1.0
Acquired/reconstructed spatial resolution (mm³)	1.6/1.0	1.6/1.0
Spatial resolution (ms (cardiac phases))	∼33 (30)	∼33 (30)
Acceleration factor (median (range))	18.7 (18.7–18.9)	14.7 (8.9–14.8)
Scan time (median (range) (min:s))	4:21 (4:14–4:21)	8:20 (6:41–8:56)
Respiratory motion	Self-gating	Navigator

*bSSFP* balanced steady-state free precession, *FA* flip angle, *FOV* field of view, *TE* echo time, *TR* repetition time, *4D* four-dimensional, *CMR* cardiovascular magnetic resonance

Data are numbers or median (range).

NnU-net was used to create time-resolved segmentations on the 4D bSSFP data that were used to create displacement maps [3], [7]. Centerlines were created for all segmentations and planes were defined at the 1) sinotubular junction (STJ), 2) the mid ascending aorta (mid-AAo), at the level of the pulmonary artery bifurcation, 3) the proximal descending at the sharpest edge between the horizontal and vertical part of the descending aorta (pDAo), and 4) the level of the diaphragm (dDAo). Additionally, time-resolved AAo volumes, from the annulus to brachiocephalic trunk, and DAo volumes, from left subclavian artery to diaphragm, were used to calculate 3D distensibility, defined by $\frac{V_{\max}-V_{\mathrm{end}-\mathrm{diastole}}}{\;V_{\mathrm{end}-\mathrm{diastole}}*\mathrm{PP}}$, where *V* is volume and *PP* is averaged pulse pressure measured by sphygmomanometer before and after the CMR exam. These measurements were also used to calculate mean arterial pressure (MAP).

Time-averaged segmentations of the 4D flow data obtained from a similar nnU-net algorithm were used to calculate peak systolic velocity and wall shear stress (WSS) and PWV [8], [9]. Mean regional peak systolic velocity and WSS were calculated in four regions: 1) from the annulus to the level to the mid-AAo plane, 2) from that point to the brachiocephalic trunk, 3) from the left subclavian artery to the level of the pulmonary artery bifurcation in the DAo, and 4) from that level to the diaphragm. The peak systolic timeframe was determined by the highest spatially averaged velocity. Because of the limited sample size, no statistical analysis was performed.

## 3 Results

### 3.1 Patient characteristics

Three patients underwent valve-sparing root surgery. Patient 1 was a 37-year-old female (body surface area [BSA] 1.95 m^2^), patient 2 a 29-year-old male (BSA 2.16 m^2^), and patient 3 a 26-year-old male (BSA 2.16 m^2^), with preoperative aortic root diameters of 46.0, 49.2, and 50.9 mm, respectively. Diameters distal to the aortic root were within the normal range. Preoperative aortic regurgitation derived from clinical echocardiography was moderate in patients 1 and 2 and mild in patient 3, with mild regurgitation remaining only in patient 1 postoperatively. No aortic valve stenosis was present before or after surgery. Patients 1 and 3 were prescribed Losartan, while patient 2 was treated with Metoprolol throughout follow-up. Previous genetic testing revealed suspected haploinsufficient *FBN1* variants in patients 1 and 2, and a suspected dominant negative *FBN1* variant in patient 3. Patients 1 and 2 underwent a David procedure, whereas patient 3, who had a bicuspid aortic valve (raphe between the left and right coronary cusp), underwent a Lansac procedure.

Patients 1 and 2 also received concomitant aortic valve repair.

MAP (mmHg) and pulse pressure (mmHg) showed small changes from baseline to follow-up: patient 1’s MAP increased from 87 to 92, pulse pressure from 42 to 48; patient 2’s MAP decreased from 93 to 89, pulse pressure from 56 to 45; patient 3’s MAP decreased from 108 to 106, pulse pressure increased from 45 to 49.

### 3.2 3D distensibility and displacement

CMR was acquired 335, 5, and 31 days before aortic root surgery, and 168, 174, and 226 days after surgery for patients 1, 2, and 3, respectively.

Video 1 shows the pre- and post-operative 3D displacement maps and maximum intensity projections of the 4D flow data for the three patients.

**Video 1:**
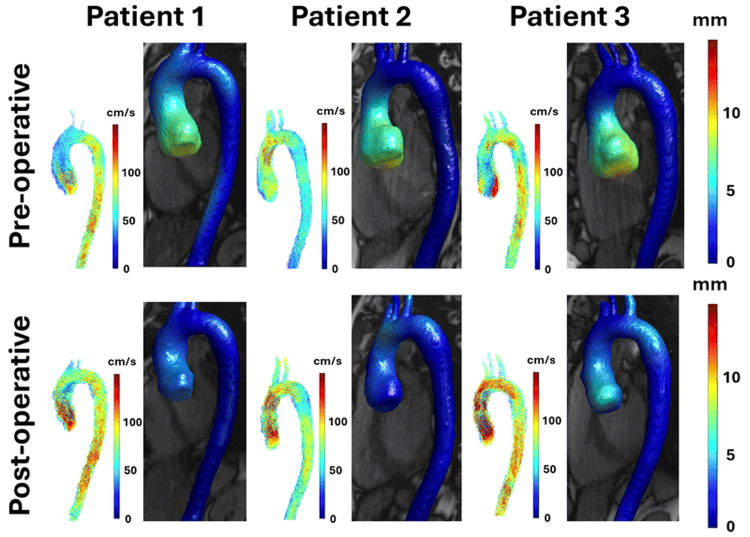
3-dimensional displacement maps and flow vectors before and after aortic root surgery. *(Single frame from video)*.

**Fig. 1:**
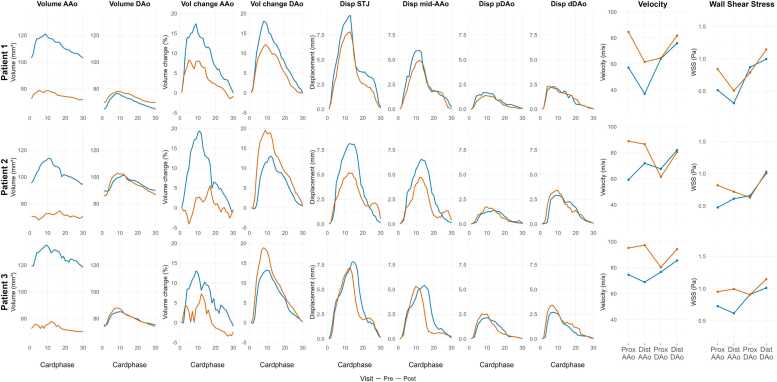
Volume, 3D distensibility, displacement derived from 3D cine bSSFP CMR, and regional mean velocity and WSS before (blue) and after (orange) aortic root replacement surgery. *AAo* ascending aorta, *DAo* descending aorta, *dDAo* distal descending aorta plane, *Dist* distal, *Disp* displacement, *pDAo* proximal descending aorta plane, *Prox* proximal, *Vol* volume, *STJ* sinotubular junction, *3D* three-dimensional, *bSSFP* balanced steady-state free precession, *CMR* cardiovascular magnetic resonance, *WSS* wall shear stress

In Fig. 1, the quantification of 3D volume (change), displacement, velocity, and WSS is presented. Changes in the AAo are most pronounced, with a markedly lower volume after surgery, with barely any volume changes during the cardiac cycle. This results in a decrease in AAo 3D distensibility (10^−3^ mmHg^−1^) in patient 1, from 4.1 to 1.7, patient 2 from 3.4 to 1.2, and patient 3 from 2.9 to 1.5. In the DAo, the changes are more subtle but still observable, with maximum aortic volumes slightly increasing in all patients. Patients 2 and 3 exhibit an increase in 3D distensibility (10^−3^ mmHg^−1^) (from 2.3 to 4.3 and 2.9 to 3.9, respectively), while patient 1 shows an increase in end-diastolic volume along with a decrease in 3D distensibility (10^−3^ mmHg^−1^): from 4.3 to 2.5.

Postoperatively, displacement decreased in all patients throughout the AAo, most markedly on the STJ level. On the pDAo and dDAo level, there was a slight increase in displacement in patients 2 and 3. However, a decrease was observed in pDAo displacement in patient 1, with unchanged displacement at the dDao level.

### 3.3 Velocity, WSS, and PWV

Velocity and WSS increased after surgery throughout the AAo, while in the DAo, parameters remained largely unchanged except for an increase in velocity and WSS in patient 3. For patient 1, PWV decreased (m/s) from 8.4 to 7.9, while in patient 2, PWV increased from 8.0 to 8.9, and in patient 3, from 6.2 to 9.8.

## 4 Discussion

In this study, we acquired 4D bSSFP and 4D flow CMR before and after aortic root surgery. These combined methods allow for a convenient side-by-side comparison of changes in aorta biomechanics and hemodynamics. We found that displacement of the AAo decreased, probably as a result of the implantation of the graft, which is presumably stiffer than the native tissue. This notion is supported by a reduction in 3D distensibility in the AAo. The graft also causes a substantial decrease in the aortic volume. It has been hypothesized that the graft is unable to absorb systolic forces in the same way as the native root, leading to undamped, turbulent flow in areas distal to graft placement [2]. In our data, we observed increased velocity and WSS throughout the AAo after surgery, but no large changes in hemodynamics were observed in the DAo. However, there were small changes in 3D distensibility and displacement in the pDAo as well as in global PWV: a decrease in patient 1 and an increase in patients 2 and 3. It is difficult to predict whether higher or lower distensibility and displacement contribute to TBAD. A decreased distensibility could indicate that tissue is not compliant enough to absorb forces, which may have consequences, whereas an increased distensibility may indicate increased forces on the tissue that might make it prone to dissection. The fact that changes were not uniform could be a result of slightly different patient profiles. Notably, there were differences in the *FBN1* gene variant, presence of a bicuspid aortic valve, aortic diameters, and medication use. Furthermore, patient 1 was a female who was ∼10 years older than the other two participants, with a longer time between both CMR examinations, and patient 3 underwent aortic root remodeling with external ring annuloplasty rather than root surgery with reimplantation of the aortic valve, which is known to preserve physiologic movements of the cusps within the three reconstructed neo-sinuses, possibly resulting in less prominent changes in aortic displacement [10].

## 5 Conclusions

Valve-sparing aortic root surgery in MFS induces measurable alterations in thoracic aortic biomechanics and hemodynamics, particularly reduced distensibility and displacement in the AAo and increased local flow velocities and WSS. These findings suggest that the stiffer graft material modifies aortic motion and flow dynamics, potentially influencing distal aortic stress. However, effects in the DAo were not consistent between patients. Comprehensive studies with a larger sample size and long-term follow-up are warranted to determine whether such biomechanical and hemodynamic changes contribute to the risk of developing TBAD in post surgical MFS patients.

## Funding

This study is part of the research program Applied and Engineering Sciences and the project Comprehensive Assessment of 4D Thoracic Aorta Biomechanics Using Novel Cardiac MRI Technology (number 18402), financed by the Dutch Research Council (NWO). E.M.S. acknowledges funding by 10.13039/100011950ITEA Eureka cluster on Software innovation through the SIGNET project number 20052.

## Author contributions

**Pim van Ooij:** Writing – review & editing, Supervision, Methodology, Funding acquisition, Conceptualization. **Maarten Groenink:** Writing – review & editing, Supervision, Methodology, Conceptualization. **Moniek G.P.J. Cox:** Writing – review & editing, Investigation. **Aart J. Nederveen:** Writing – review & editing, Supervision, Conceptualization. **Dave R. Koolbergen:** Writing – review & editing, Investigation. **Daniëlle Robbers-Visser:** Writing – review & editing, Supervision. **Eric M. Schrauben:** Writing – review & editing, Supervision, Software, Investigation, Funding acquisition, Conceptualization. **Renske Merton:** Writing – review & editing, Methodology, Investigation. **Daan Bosshardt:** Writing – original draft, Visualization, Project administration, Methodology, Investigation, Formal analysis, Conceptualization.

## Declaration of Generative AI and AI-assisted technologies in the writing process

During the preparation of this work, the authors used Grammarly and ChatGPT to correct English grammar/spelling and improve readability. After using this tool/service, the authors reviewed and edited the content as needed and take full responsibility for the content of the publication.

## Declaration of competing interests

The authors have no competing interests to declare.

## Data Availability

For this study, we used the Amsterdam UMC “PROspective Undersampling in multiple Dimensions” (PROUD) software patch (https://mriresearch.amsterdam/software/aumcproudpatch/). All data and software are available on reasonable request.
