# Supplementary figures and images for: Assessing changes in aortic motion and hemodynamics after valve-sparing aortic root surgery in Marfan syndrome using four-dimensional balanced steady-state free precession and four-dimensional flow cardiovascular magnetic resonance
Source: J Cardiovasc Magn Reson. 2026 Apr 17;28(1):102728. doi: 10.1016/j.jocmr.2026.102728 (PMC13254676; doi:10.1016/j.jocmr.2026.102728)

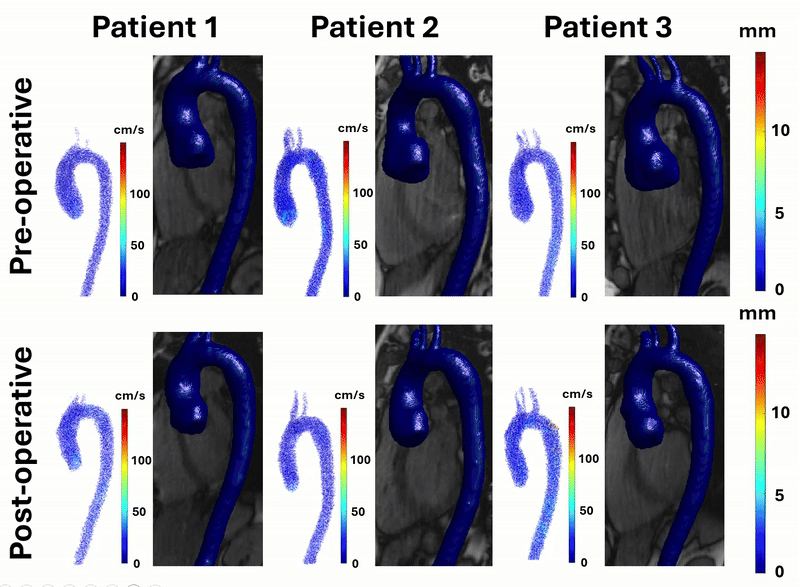

Supplement: Supplementary file 1 [file mmc1.gif]
